# Macrophages and the maintenance of homeostasis

**DOI:** 10.1038/s41423-020-00541-3

**Published:** 2020-09-15

**Authors:** David M. Mosser, Kajal Hamidzadeh, Ricardo Goncalves

**Affiliations:** 1 The Department of Cell Biology and Molecular Genetics, The University of Maryland, College Park, MD 20742 USA; 2 The Department of General Pathology, Federal University of Minas Gerais, Belo Horizonte, MG Brazil

**Keywords:** Macrophages, polarization, cytokines, development, inflammation, Leukopoiesis, Cytokines

## Abstract

There have been many chapters written about macrophage polarization. These chapters generally focus on the role of macrophages in orchestrating immune responses by highlighting the T-cell-derived cytokines that shape these polarizing responses. This bias toward immunity is understandable, given the importance of macrophages to host defense. However, macrophages are ubiquitous and are involved in many different cellular processes, and describing them as immune cells is undoubtedly an oversimplification. It disregards their important roles in development, tissue remodeling, wound healing, angiogenesis, and metabolism, to name just a few processes. In this chapter, we propose that macrophages function as transducers in the body. According to Wikipedia, “A transducer is a device that converts energy from one form to another.” The word transducer is a term used to describe both the “sensor,” which can interpret a wide range of energy forms, and the “actuator,” which can switch voltages or currents to affect the environment. Macrophages are able to sense a seemingly endless variety of inputs from their environment and transduce these inputs into a variety of different response outcomes. Thus, rather than functioning as immune cells, they should be considered more broadly as cellular transducers that interpret microenvironmental changes and actuate vital tissue responses. In this chapter, we will describe some of the sensory stimuli that macrophages perceive and the responses they make to these stimuli to achieve their prime directive, which is the maintenance of homeostasis.

## Introduction

Macrophages were originally described by Metchnikoff^1^ during his studies of primitive animals devoid of adaptive immune mechanisms. The cells were called phagocytes, from Greek—phagein (to eat) and cytes (cells). The process of phagocytosis was initially recognized to be involved in the homeostatic processes of tissue resorption and the acquisition of nutrients. Subsequently, Metchnikoff^1^ deduced that this process could also be used to protect our body against invaders. In no small way did the prescient observations of Metchnikoff^2^ on the phenomenon of phagocytosis form the foundation for our present-day understanding of cellular immunity against microbes. Thus, the initial concept of macrophages promoting “balance” in the host (homeostasis) was largely ignored and essentially overshadowed by the involvement of macrophages in cellular immune responses.

**Fig. 1:**
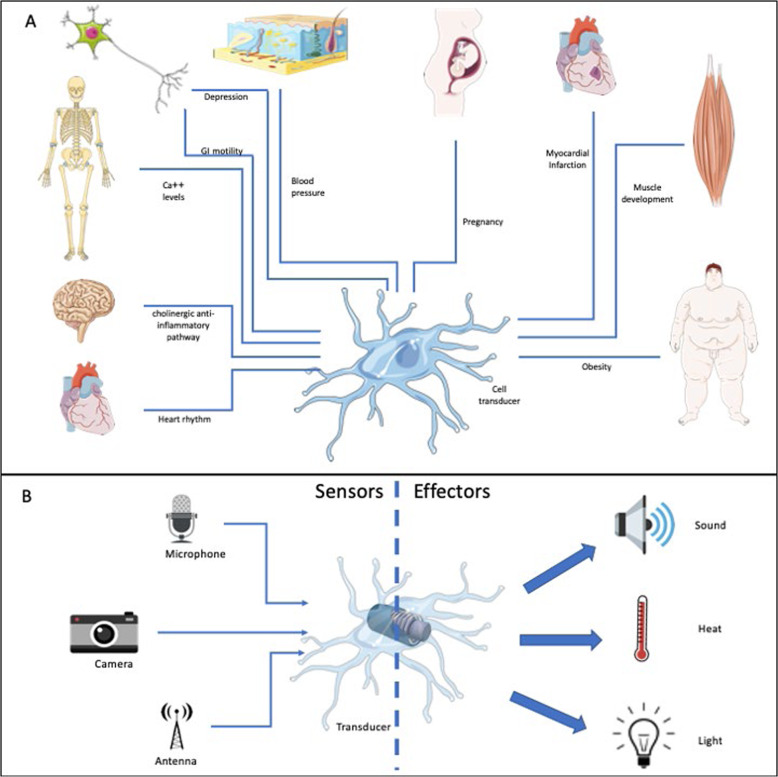
Macrophages as transducers. An analogy was made regarding macrophages functioning as a transducer of information to generate a model. **a** Macrophages from different tissues function by collecting information from the microenvironment, processing this information, and transducing it to generate important chemical responses for the specific functioning of an individual organ. **b** Different stimuli or combinations of stimuli, which are depicted as different forms of energy, are received by macrophages and transduced into different outputs. This image was prepared using Medical ART (https://smart.servier.com/)

Macrophages are present in virtually all tissues in the body, where they maintain proper organ function. They are involved in the metabolism of iron, bilirubin, calcium, lipids, and amino acids, and contribute to the maintenance of fairly constant levels of these substances in the body.^3–7^ Most of these homeostatic functions are related to efferocytosis, which is a primitive process identified in starfish more than 100 years ago. Macrophage phagocytosis allows the removal and recycling of enormous numbers of dead cells and tissue debris, which would prevent organ function if they were allowed to accumulate. This type of clearance occurs in all organisms and proceeds unperturbed in the absence of adaptive immune responses and, in some cases, in the absence of blood. The contributions of tissue macrophages to healing were originally described by Metchnikoff^1^ in invertebrates that lacked blood. However, in early embryos in higher vertebrates, prior to the development of blood vessels, tissue macrophages can contribute to the process of healing and tissue regeneration. Macrophages play important roles in the steady state, in which they are typically the only tissue-resident “immune” cells. Macrophages resident in the eyes,^8^ joints,^9^ mammary glands,^10^ and ovaries^11^ maintain tissue integrity by integrating input signals from tissues and conveying instructions to neighboring stromal cells. Macrophage-mediated homeostasis is so important that macrophages are even present in human breast milk, where they may contribute to the control of the digestive tube balance in the infant.^12^ They also exert regulatory functions with important roles in the control of inflammation and the promotion of healing responses in newborns.^13^ Indeed, macrophages are the only cells present in every organ in the body. Macrophages are present in the epidermis, cornea, and the insides of joints, where blood vessels do not exist. In this context, macrophages are vital cells that function as transducers by obtaining information from the tissues and translating it to induce reactions. These reactions are typically related to the physiological functions that are essential for the day-to-day operation of that organ (Fig. 1a).

Macrophages have been described in many different situations as cells capable of sensing the microenvironment and responding to the needs of the organ. As Metchnikoff stated, macrophages are always seeking balance but are also helping the organ to perform its unique individual functions. During pregnancy, e.g., they sense the signals from hormones and transduce them to establish an immune-tolerant environment to allow the embryo to develop in an environment where it would otherwise be rejected. They also help to build the placenta, without which there would be no fetal development.^14^ In the heart, macrophages interact with cardiomyocytes, sensing them and accelerating their repolarization, and thereby maintaining cardiac conduction. The depletion of macrophages induces progressive atrioventricular block.^15^ Interesting recent work has demonstrated that macrophages can transduce osmotic signals from tissues and can control blood pressure in mice and rats fed a high-salt diet.^16^ In fact, macrophages are able to recognize signals resulting from NaCl hypertonicity and migrate in the direction of a high salt concentration.^17^ These are just a few examples to illustrate the most important function of these cells, which is to maintain homeostasis. By functioning as transducers, macrophages interpret their microenvironment and provide instructions to neighboring cells to maintain balance (Fig. 1b). These “outputs” are much more complex than the inflammatory cytokines that have been associated with the stimulation of macrophages. They can include matrix metalloproteinases, which remodel the extracellular matrix, vascular endothelial growth factor (VEGF) and thrombospondin, which induce angiogenesis, and growth factors, which promote wound healing. In this context, it is a gross oversimplification to consider macrophages merely as “immune” cells.

The capacity of macrophages to sense their environment is not just a theoretical possibility but can be exploited to improve the diagnosis and therapy of diseases, such as cancer. One of the major challenges in treating cancer is the detection of early metastasis. Gulati and colleagues^18^ recently developed an engineered macrophage reporter that expresses luciferase under the control of the arginase-1 promoter. Thus, when this cell is attracted to the tumor microenvironment, it becomes a macrophage with a tumor-associated profile (tumor-associated macrophage) and begins to express luciferase, which can be easily detected to indicate possible metastasis.^18^ As we have come to better understand the transducer functions of macrophages, we have realized that the simple M1/M2 classification of macrophages simply does not reflect reality, and that these cells can be manipulated in predictable ways simply by changing the input stimuli.

## Macrophage origins

There has been much recent interest in the ontogeny of macrophages following the seminal observations of several groups,^19–27^ revealing that yolk-sac-derived tissue-resident macrophages are largely maintained independently of hematopoietic input. These observations have given rise to the idea that newly migrated monocyte-derived macrophages are inflammatory in nature, whereas tissue-resident cells function to mitigate inflammation and restore homeostasis.^28–30^ This is an attractive idea that is consistent with some good experimental data.^29,31–33^ However, this idea is inconsistent with what we consider to be a fundamental property of macrophages, which is plasticity. All macrophages, including tissue-resident cells, express pattern recognition receptors that allow them to respond to activating stimuli. Thus, all macrophages, including yolk-sac-derived tissue-resident macrophages, can respond to the various stimulatory inputs that are described in subsequent sections of this chapter. Furthermore, all macrophages exhibit plasticity in their capacity to change their phenotype in response to changes in their microenvironment. Thus, all macrophages have the potential to respond to adenosine, PGE2, or other modulating molecules produced in tissue that dampen inflammatory responses and skew responses toward tissue repair. There may be an alternative way to interpret some of the anti-inflammatory responses that have been attributed to tissue-resident macrophages. In our opinion, there are likely two major tissue-derived influences that bias resident tissue macrophages toward an immune resolution phenotype. First, during the steady state, tissue-resident macrophages are likely exposed for prolonged periods of time to tissue-derived modulators that promote cell growth to maintain normal tissue development. These modulators can include adenosine, PGE2, and the resolvins, which are described below. They are also exposed to apoptotic cells, which can modulate their phenotype.^34^ Second, the macrophage growth factor, macrophage colony-stimulating factor (M-CSF), is produced by many cells to bias macrophages toward a growth-promoting and angiogenic phenotype (Hamidzadeh et al., in press). During the steady- state M-CSF predominates, whereas during inflammation granulocyte M-CSF is transiently produced to promote inflammatory responses by all macrophages exposed to it. Thus, the inputs and responses we describe below are germane to all macrophages, regardless of their origins.

## Sensory inputs that change macrophage physiology

### Pathogen- and damage-associated molecular patterns

Metchnikoff believed that there could be no cure without inflammation.^35^ We now know a great deal about inflammation and the molecules that participate in it. We also know that in addition to initiating curative processes, inflammation must be tightly regulated, because uncontrolled inflammation can lead to tissue pathology. It is the regulation of inflammation that this chapter is focused on. To start, however, some mention of the pathogen- and damage-associated molecular patterns (PAMPs and DAMPs) that initiate inflammation is warranted. In the simplest terms, macrophages are endowed with a large assortment of receptors that recognize molecular patterns that are portents of danger. They undergo profound alterations in gene expression in response to receptor binding of these signals. Human macrophages exposed to nanomolar concentrations of bacterial lipopolysaccharide (LPS), e.g., differentially express some 4500 genes compared to resting macrophages.^36^ This change in gene expression gives rise to what have been referred to as M1 macrophages, which are inflammatory in nature (note: the term “classically activated macrophages” is typically reserved for antimicrobial macrophages exposed to toll-like receptor (TLR) ligands and the cytokine interferon (IFN-γ). Even very small perturbations in the steady state will give rise to a population of stimulated inflammatory (M1) macrophages. These cells express a variety of inflammatory cytokines and chemokines. The induction of these inflammatory responses is carefully balanced by a variety of negative regulators of TLR signaling. These regulators can work to inhibit transcription factors, adaptor complexes, signaling pathways, and receptor ligand binding itself. For a review of these regulators, see ref. ^37^.

### Cytokines

There is no debate as to whether macrophages are exquisitely sensitive to cytokines produced in their local microenvironment. The two most extensively studied cytokines affecting macrophage physiology are IFN-γ and interleukin 4 (IL-4). In fact, these two cytokines exert such dramatic and different influences on macrophage physiology that macrophages have been described as M1 and M2 based on their exposure to either one of these cytokines. Macrophages express high-affinity receptors for IFN-γ^38^ and, in response to IFN-γ, they undergo myriad changes in gene expression to become the potent antimicrobial cells that Mackaness,^39^ Cohn and colleagues,^40^ and Nathan et al.^41^ originally described. In simplified terms, cell-mediated immune responses are mounted to produce IFN-γ, to activate macrophages and kill intracellular organisms.

The role of IL-4 in macrophage physiology has also been extensively studied^42,43^ but somehow remains less well defined. This T_H_2 cytokine, which is primarily produced in response to helminthic infections and allergic reactions,^43^ exerts a profound effect on macrophages, causing them to assume a fundamentally different activation state, which was originally termed the “alternatively activated” state.^44^ There are two major points of confusion regarding macrophage responses to IL-4. The first pertains to biomarkers that identify alternatively activated macrophages (AA-Mϕ). In the murine system, the response of macrophages to exogenous IL-4 is quite dramatic. In our hands, within 4 h of IL-4 administration, activated murine macrophages upregulated 23 transcripts by 25-fold or more, compared to resting macrophages (Table 1). These upregulated genes included Ym1 (Chitinase-like 3), Retnla (Fizz1, RELMα), and Mrc1, which is the mannose receptor that was originally used to identify AA-Mϕ.^44^ The use of a combination of these biomarkers (preferably all three) provides a confident identification of murine IL-4-treated AA-Mϕ. Human IL-4-treated macrophages are not as easy to identify. Several of the transcripts expressed by murine IL-4-treated macrophages are unique to mouse macrophages, including those of the chitinases that are associated with murine AA-Mϕ. The extent to which IL-4 influences human macrophage gene expression also appears to be more modest, making biomarker identification more difficult. In our hands, only one of the top 23 murine IL-4-induced genes was extensively upregulated in human macrophages. Thus, the identification of human AA-Mϕ in tissue is not a trivial exercise and many groups have misidentified these cells using murine markers that simply do not pertain to human AA-Mϕ. The second major point of confusion regarding the so-called M2 macrophages is the role of IL-4 in wound healing. In a series of elegant papers, Wynn and colleagues^45–48^, working in the murine system, described the induction of arginase expression in IL-4-treated macrophages. As arginase converts arginine to ornithine, a precursor of polyamine biosynthesis, these murine IL-4-treated macrophages were associated with wound-healing responses. Subsequent to these studies, many groups have tentatively identified M2 macrophages as the cells that promote wound healing. The extent to which IL-4 contributes to wound healing and whether IL-4 is required to produce wound-healing macrophages appears to be an issue associated with some confusion in macrophage biology. At first glance, it might not make sense that a fundamental healing response would be so dependent on a single cytokine produced primarily by adaptive immune cells, as is IL-4. In fact, there are many experimental examples of wound healing that occur independently of adaptive immune responses. For example, wound healing occurs normally in SCID mice, which lack mature T cells. The production of granulation tissue can actually be increased without the influence of lymphocytes.^49^ Wound-healing macrophages can be observed in IL-4R-knockout mice and the wound-healing macrophage phenotype did not require IL-4 or IL-13.^50^ More recent studies have shown the involvement of other pathways in the transition of M1 macrophages into M2 macrophages, which are independent of IL-4 and IL-13.^51^ Below, we provide some examples in which macrophages promote tissue regeneration in the absence of any obvious contribution of the immune cytokine IL-4.

**Table 1:** Gene expression induced by IL-4 treatment of mouse macrophages

Symbol	Name	Log FC_2_	Adj. *P*-value	Human (FC_2_)*
Chil3**	Chitinase-like 3 (Ym1)	8.7	9.1^−^^13^	x
Cd209e	CD209e antigen	6.9	1.9^−6^	3.16
Itgb3	Integrin beta 3	6.8	7.8^−9^	-
Ear11	Eosin-associated ribonuclease A	6.3	8.5^−6^	-
Flt1	FMS tyrosine kinase	6.3	4.9^−7^	-
Serpina3g	Serine peptidase inhibitor	6.21	6.2^−7^	-
Chi3l4	Chitinase 3-like 4	5.9	6.5^−6^	-
Pdcd1lg2	Programmed cell death 1 ligand 2	5.7	2.3^−10^	1.25
Slc7a2	Solute carrier family 7	5.7	3.3^−8^	-
Cdh1	Cadherin 1	5.4	1.8^−7^	-
Ntrk1	Neurotrophic TKR type 1	5.4	1.9^−4^	-
Tmem26	Transmembrane protein 26	5.3	7.8^−4^	2.72
Tslp	Thymic stromal lymphopoietin	5.3	1.0^−4^	-
Il4i1	IL-4-induced 1	5.2	1.9^−9^	-
Il31ra	IL-31 receptor A	5.1	1.0^−4^	-
En2	Engrailed 2	5.0	7.8^−6^	-
Cish	Cytokine-induced SH2	5.0	7.7^−6^	4.79
Mrc1**	Mannose receptor	5.0	3.6^−7^	2.37
Apo7c	Apolipoprotein L 7c	5.0	1.8^−6^	x
Socs1	Suppressor cytokine signaling	5.0	7.7^−8^	3.45
Retnla**	Resistin-like alpha (Fizz1)	4.8	1.6^−9^	x
Ccl7	Chemokine (CC) ligand 7	4.6	4.3^−3^	-
Ccl12	Chemokine (CC) ligand 12	4.6	4.6^−4^	-

*Fold change in human macrophages treated with IL-4; “x” designates genes that are specific to mice and not found in humans.

**Bold symbols designate the three most widely used biomarkers for murine M2a macrophages.

Our intention is not to minimize the contribution of cytokines to macrophage biology. In fact, macrophages have receptors for many different cytokines. It is likely that macrophages in tissue encounter a combination of cytokines, making their responses far more complex than the original M1/M2 designation suggested. For example, IL-4 alone does not enhance tumor necrosis factor (TNF) secretion by macrophages. However, in combination with IL-33, these two cytokines are able to induce production of both soluble and membrane-bound TNF, as well as IL-6 by macrophages.^52^ IL-6 can enhance the expression of markers of AA-Mϕ in mice, but in combination with IFNγ it increases the production of the proinflammatory cytokines IL-1β and TNF.^53^ Cytokines modulate many macrophage functions and it should be appreciated that macrophages themselves can be their own source of cytokines. Autocrine IL-33 signaling can lead to the upregulation of matrix metalloproteinases, including MMP2 and MMP9, in rat alveolar macrophages.^54^ Much of the tumoricidal activity of LPS-stimulated macrophages is dependent on autocrine IFNα and IFNβ signaling.^55^ Autocrine IL-10 production by tumor-associated macrophages reduces the capability of these macrophages to produce inflammatory IL-12.^56^ IL-10 has been well-characterized in terms of its anti-inflammatory programming of macrophages, but it is not the only regulatory cytokine.^57,58^ Low levels of autocrine IL-15 suppress macrophage proinflammatory cytokine production.^59^ IL-21 inhibits the LPS-induced expression of the proinflammatory cytokines IL-1β, TNF, and IL-6 in mouse peritoneal macrophages.^60^ IL-35 activates transforming growth factor (TGF)-β in macrophages and promotes their function in wound healing by inducing extracellular matrix deposition.^61^ Therefore, cytokines can both stimulate macrophage inflammatory responses, and dampen inflammation and promote tissue repair functions.

Many important macrophage functions proceed normally in the absence of cytokines associated with adaptive immune responses. Macrophages play a well-described role in muscle development.^62^ During weight training, concentric contraction results in damage to the muscle fiber. Macrophages migrate into the muscle to clear tissue debris. They assume an inflammatory phenotype in response to the DAMPs released from dead and dying cells. The sore muscles that arise following weight training are the result of this process. Muscle macrophages then undergo a physiological change that allows them to signal to satellite cells, which are muscle-resident stem cells, to divide and differentiate into new muscle fibers. These new muscle fibers provide added strength to the muscle following recovery. We are not aware of reports of lymphocyte migration into the developing muscle and, as far as we know, experimental animals with genetic deletion of T and B cells undergo normal muscle development.^63,64^ Thus, unless there is some unknown innate source of IL-4 release from damaged muscle during exercise, we cannot determine the definitive role of IL-4 in macrophage-mediated promotion of muscle development. A similar repair process may occur during recovery from cardiovascular disease. Macrophages migrate into the heart in response to myocardial injury and commence to promote the clearance of necrotic tissue. Subsequent to this, they switch their phenotype to promote stem cell differentiation and healing. Cardiac macrophages are now thought to participate in tissue remodeling and self-renewal of cardiac tissue.^65^ As self-reactive T cells have been clonally deleted during development, the contribution of antigen-specific lymphocytes has been deemed to be minimal. Thus, the process of wound healing in the heart occurs in the absence of IL-4 and without the contribution of adaptive immunity.

### Immune complexes

Immune complexes (ICs) are sensory stimulators that exert a profound influence on macrophage physiology. When macrophages are stimulated in the presence of high-density ICs, they downregulate the production of multiple inflammatory mediators and upregulate the production of anti-inflammatory cytokines, as well as growth-promoting and angiogenic factors. Thus, stimulation in the presence of ICs induces a dramatic change in the physiology of macrophages relative to stimulation in the absence of ICs. When these changes in gene expression were originally observed,^66–69^ they were somewhat difficult to interpret within the background of the well-accepted importance of IgG in promoting antigen-specific immune responses. We now think that macrophages alter their physiology in response to stimulation in the presence of ICs to terminate humoral immune responses and initiate the necessary tissue repair processes. As all immune responses have the potential to cause tissue damage, they must all be accompanied by homeostatic regulation to initiate healing responses. Thus, IgG antibody responses are produced in response to foreign antigens. ICs cross-link Fcγ receptors to initiate phagocytosis, but macrophages interpret ICs as a signal to terminate immune responses and initiate tissue repair.

This response to ICs may prevent an overzealous humoral immune response from causing too much tissue damage, but it can also be detrimental to the host. In leishmaniasis, high numbers of ICs have been associated with defective macrophage killing of intracellular parasites. In the most severe form of cutaneous leishmaniasis, called diffuse cutaneous leishmaniasis, parasites replicate uncontrolled in dermal macrophages that are literally swimming in a sea of IgG ICs.^70^ This may be due to the ability of ICs to signal to macrophages to dampen inflammatory responses and promote a wound-healing response. Respiratory viruses may also take advantage of this alteration in macrophage physiology. Dengue virus has been associated with antibody-dependent enhancement of disease following the generation of humoral antiviral responses.^71^ Respiratory syncytial viruses and some feline coronaviruses have been associated with a phenomenon called vaccine-associated enhanced respiratory disease, which can develop after humoral immune responses.^72^ It is unclear whether the alteration of macrophage phenotypes in response to the binding of FcγR by viral ICs may contribute to the exacerbation of forms of respiratory disease.

### Endogenous regulators

**Fig. 2:**
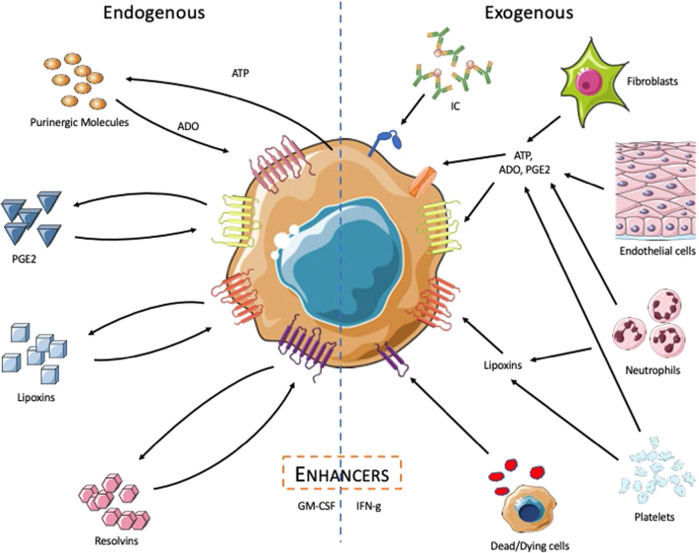
Macrophage responses to endogenous and exogenous regulators. Left: stimulated macrophages produce and release ATP. The macrophage ectoenzymes CD39 and CD73 rapidly convert ATP to adenosine,^47^ which signals through high-affinity receptors for adenosine to switch off the production of inflammatory mediators and to induce the production of growth-associated and angiogenic factors. Stimulated human macrophages also upregulate the synthesis of PGE_2_ and the receptors for PGE_2_, causing them to become exquisitely sensitive to the regulatory effects of PGE_2_. Macrophages also catabolize lipids into lipoxins and resolvins to dampen inflammatory responses. Right: neighboring cells, including fibroblasts, endothelial cells, neutrophils, and platelets, can also be a source of these regulatory molecules. These neighboring cells can also be stimulated to produce “enhancer” molecules that amplify the initial activation response. This image was prepared using Medical ART (https://smart.servier.com/)

Macrophages themselves produce numerous molecules that function in the maintenance of homeostasis (Fig. 2). In general, these molecules are produced in response to an activating signal, such as TLR stimulation, which in addition to promoting inflammatory responses also activates regulatory mechanisms to limit inflammation and restore homeostasis. This is an important process because prolonged inflammation can lead to tissue destruction. The contribution of a few endogenous regulators, including adenosine, prostaglandin E2 (PGE_2_), resolvins, and lipoxins, to the active resolution of inflammation will be discussed.

Adenosine is a purine nucleoside that is derived from endogenously produced ATP.^73^ ATP released by macrophages in response to stress is rapidly converted into adenosine at the cell surface by the ectoenzymes CD39 and CD73.^74^ Adenosine is sensed via four G-protein-coupled receptors, A1r, A2Ar, A2Br, and A3; two of these, A2Ar and A2Br, lead to increased intracellular cAMP levels via Gαs signaling.^75^ A2Ar and A2Br signaling potentiate the regulatory effects of adenosine by dampening inflammatory cytokine production and increasing IL-10 production by macrophages.^76–78^ Adenosine sensing also leads to the production of VEGF.^79–81^ VEGF is an important growth factor for angiogenesis and wound healing.^82^

PGE_2_ is an endogenous lipid derived from arachidonic acid in the plasma membrane. PGE_2_ is the most widely acting and most studied prostaglandin. It is sensed by macrophages via four G-protein-coupled receptors, EP1, EP2, EP3, and EP4, at the cell surface.^83^ PGE_2_ is produced by macrophages in response to PAMPs and upon uptake of apoptotic cells.^34,84,85^ It is widely thought that a switch from prostaglandin and leukotriene synthesis to the production of resolvins and lipoxins marks the pro-resolution phase of inflammation.^86^ However, it should be appreciated that in the context of macrophage activation, PGE_2_ induces anti-inflammatory responses and plays an active role in the restoration of homeostasis.^87^ PGE_2_ sensing leads to the suppression of inflammatory cytokine release by macrophages, including TNF, IL-1β, and IFNβ.^88–90^ Most of the anti-inflammatory effects of PGE_2_ are produced via the EP2 and EP4 receptors, which lead to intracellular cAMP release.^91–93^ PGE_2_ has been demonstrated to play a role in skeletal muscle regeneration.^94^ In addition, macrophage-derived PGE_2_ was shown to play a critical role in the resolution of inflammation at sites of tissue injury by affecting neutrophil recruitment.^95^

Resolvins are another family of lipid mediators that contribute to the homeostatic role of macrophages. Resolvins are derived from the omega-3 polyunsaturated fatty acids, eicosapentaenoic acid and docosahexaenoic acid, and are called E-series and D-series resolvins, respectively.^96^ To date, the known receptors for resolvins are the G-protein-coupled receptors GPR32, GPR18, and ChemR23.^97^ Human macrophages were shown to produce the resolvins D2 (RvD2) and D5 (RvD5) in response to *Escherichia coli* and *Staphylococcus aureus* stimulation.^98^ Resolvin D1 (RvD1) was shown to signal through its receptor GPR32 on macrophages to decrease IL-1β and IL-8 secretion, reduce chemotaxis, and increase phagocytosis.^99^ RvD2 was able to inhibit NLRP3 inflammasome activation in mouse macrophages.^100^ RvD1 and RvD2 were also demonstrated to decrease the levels of IL-6 and TNF produced by human alveolar macrophages and to increase the levels of TGFβ produced by human monocyte-derived macrophages.^101^

Lipoxins are yet another class of lipid mediators derived from arachidonic acid that contribute to the resolution of macrophage activation. LXA_4_ and LXB_4_ are lipoxins produced by the lipoxygenase (LO) enzymes 15-LO, 5-LO, and 12-LO.^102^ Their stable aspirin-triggered counterparts AT-LXA_4_ and AT-LXB_4_ are produced by COX-2 in the presence of aspirin.^103^ ALX/FPR2 is a G-protein-coupled receptor for LXA_4_ and AT-LXA_4_.^104^ LXA_4_ has been shown to be produced at picogram levels by human alveolar macrophages and these levels are increased in response to LPS stimulation.^105,106^ LXA_4_ decreases TNF levels in response to LPS in macrophages by reducing the phosphorylation of IκB and nuclear factor-κB.^107^ LXA_4_ was also shown to inhibit reactive oxygen species production and granulocyte CSF (G-CSF) secretion in RAW264.7 macrophages.^108^

The endogenously produced molecules described above contribute to the transient nature of macrophage activation by dampening the production of inflammatory cytokines. At the same time, they reprogram macrophages to become coordinators of the tissue repair process. For example, one function of resolvins, specifically RvD2, is to terminate the infiltration of polymorphonuclear (PMN) cells into infection sites and to increase phagocytosis, which presumably serves to clear the infection site of debris and apoptotic cells.^109,110^ Lipoxins also trigger macrophages to increase their phagocytosis of apoptotic PMN cells.^111^ Exogenous sources of adenosine, PGE_2_, resolvins, and lipoxins can be sensed by macrophages and cause them to alter their phenotype. Tumors produce adenosine and PGE_2_, which contribute to the anti-inflammatory and proangiogenic phenotype of tumor-associated macrophages.^112,113^ Fibroblasts and endothelial cells are also sources of exogenous adenosine and PGE_2_, which can shape macrophage responses.^114–118^ Platelets and neutrophils are major sources of lipoxins.^119,120^ Therefore, macrophages can transduce signals received from different sources in the tissue environment to promote homeostasis.

### Macrophages and development

Macrophages derived from progenitors of the yolk sac^27^ appear in the embryo early in development. They play an important role in embryogenesis. During development, some tissues need to be reconstructed to assume their eventual shape once the fetus is fully formed. This reconstruction involves the removal of cells to open up new spaces or to modify the size and shape of the organ. This reconstruction is largely accomplished by programmed cell death and removal of apoptotic cells by macrophages.^121^ One of the best examples of this is the individualization of digits during development. Interdigital cell death allows the separation of digits that are joined by interdigital membranes during embryonic development.^122^ Macrophages are indispensable cells for the reabsorption of cellular debris during this process and the promotion of tissue reorganization. Alterations in macrophage function during embryogenesis can lead to defects in the development of tissues and organs,^123^ but mice lacking B and T cells exhibit no apparent defects in development. An important review about trophic macrophages indispensable for the development of several different tissues has been published.^124^ The removal of apoptotic cells by macrophages has been associated with a phenotype that not only suppresses inflammation but also promotes the development of tolerance to self-antigens.^125^ These phenotypic changes to macrophages during development do not depend on T-cell cytokines.

### Nervous system inputs

Macrophages play an important role in the development and maintenance of the central nervous system (CNS).^126,127^ These cells include not only microglia but also so-called CNS-associated macrophages. Macrophages promote the differentiation and survival of neurons and the maintenance of neuronal function through the secretion of trophic factors that are fundamental for life. The lack of macrophage control in the CNS has been associated with psychosomatic diseases, such as depression and inflammatory bowel diseases.^128^ A seminal paper called “The macrophage theory of depression”^129^ has been revisited in recent reviews.^130^ In 2000, Tracey and colleagues^131^ demonstrated that vagal stimulation could inhibit the production of proinflammatory cytokines, and they identified a “cholinergic anti-inflammatory pathway”. Shortly thereafter, Tracey^132^ wrote an important review about how the nervous system regulates inflammatory responses to control inflammation in a reflexive way. This work reinforces the participation of macrophages as transducers of the interaction with the nervous system. More recently, Chiu et al.^133^ demonstrated that bacteria can directly stimulate nociceptor sensory neurons, and that there is extensive crosstalk between nociceptor neurons and immune cells. The release of neuropeptides such as CGRP, galanin, and somatostatin can inhibit TNF transcription.^133^ An important relationship exists between macrophages and the nervous system in regulating gastrointestinal motility.^134^ Macrophages were shown to be closely positioned along nerve fibers, where they provide continuous signaling to neurons to control gastrointestinal motility. Furthermore, neurons play a key role in the maintenance of macrophage homeostasis.^134^ De Schepper et al.^135^ described a population of gut macrophages that are important to the maintenance of homeostasis, as they control several important functions associated with the vasculature, enteric neurons and intestinal motility. Depletion of these macrophages led to vascular leakage, impaired secretion, and reduced intestinal motility.^135^ Recent studies have demonstrated that intestinal infections can lead to a rapid loss of neurons and that macrophages can play a neuroprotective role in preventing neuronal cell death via β2-adrenergic receptor signaling.^136^

## Conclusion

In this work, we put forth the notion that macrophages function as transducers. These cells are vital for the development and function of virtually every organ in the body, and they are present early in embryonic development. In their role as transducers, they can sense many different endogenous or exogenous signals in tissues and rapidly respond to them. Therefore, classifying macrophages as M1 or M2 is simply not appropriate. It not only implies that they respond only to cytokines but also implies that they respond to only two cytokines! We prefer the viewpoint of Metchnikoff in that the prime function of macrophages is to achieve “balance” or homeostasis. As inflammation represents a major departure from balance, one of the major roles of macrophages is to inhibit inflammation and promote repair. This regulatory role of macrophages somehow remains underappreciated. These cells are indeed special. Their plasticity allows them to respond to myriad changes in the environment to meet the physiological needs of a particular moment in time. By better understanding the sensory inputs that macrophages perceive and the resulting physiological responses they make, we are better positioned to therapeutically manipulate these cells for the treatment of diseases.
